# The Population Ecology of Technology: An Empirical Study of US Biotechnology Patents from 1976 to 2003

**DOI:** 10.1371/journal.pone.0169961

**Published:** 2017-01-12

**Authors:** Ad van den Oord, Arjen van Witteloostuijn

**Affiliations:** 1 Tilburg School of Governance, Economics and Management, Tilburg University, Tilburg, the Netherlands; 2 Antwerp Management School, University of Antwerp, Antwerp, Belgium; 3 Cardiff Business School, Cardiff University, Cardiff, Wales; Shanxi University, CHINA

## Abstract

A detailed understanding of technological change as an evolutionary process is currently not well understood. To increase our understanding, we build upon theory from organizational ecology to develop a model of endogenous technological growth and determine to what extent the pattern of technological growth can be attributed to the structural or systemic characteristics of the technology itself. Through an empirical investigation of patent data in the biotechnology industry from 1976 to 2003, we find that a technology’s internal (i.e., density and diversity) ecological characteristics have a positive effect on its growth rate. The niche’s external characteristics of crowding and status have a negative effect on its growth rate. Hence, applying theory from organizational ecology increases our understanding of technological change as an evolutionary process. We discuss the implications of our findings for the study of technological growth and evolution, and suggest avenues for further research.

## Introduction

Although the Schumpeterian [1] conception of technological change as an evolutionary process has been widely adopted in the literature, an in-depth understanding of what this precisely entails (and does), is still argued to be in its infancy, at best [2]. If so, this implies that a great challenge is to specify a really evolutionary process that explains how technological change comes about endogenously. The purpose of the current paper is to contribute to an explanation of the nature of the growth pattern that is associated with endogenous technological change. According to [3], “Most of the recently developed endogenous growth models follow [4] and [5] in modeling technology as a scalar, pre-multiplier in an aggregate production function or as a single variable within that function.” In this paper, we take a completely different approach, and draw from organizational ecology [6] to develop a so-called ‘population ecology of technology’.

In line with [7] and [8], we argue that the notion of a technological niche offers a platform from which we can develop a deeper understanding and explanation of this process of endogenous technological growth. We define technology as a system that can be viewed as a set of interdependent technological niches or components. In turn, these niches or components are defined as populations or sets of related technological inventions. Our key aim is to develop a theory of why growth rates differ across technological components due to the structural or systemic characteristics of technology. This process of endogenous technological growth is determined by the ‘ecological’ characteristics of a technological component, and the way in which this component is embedded in the technological system and environment. This makes the concept of a technological niche useful by pointing to the important role of the structural or systemic characteristics internal to the technology in driving the process of technological growth.

The theoretical claim that this paper makes is as follows. To come to a better understanding of the process of technological growth, we argue that a systemic perspective towards technology is warranted, bringing in insights from organizational ecology. Furthermore, these technological growth patterns are to a large extent determined by structural characteristics of technology. The contribution of this paper is twofold. First, we extend the notion of the technological niche by adding internal diversity as a key structural feature. In so doing, we illustrate the importance of technological diversity in evolutionary and ecological models of technological growth. Second, we demonstrate that technology should effectively be studied as an ecological system, composed of interdependent and interacting technological components. To fully understand technological growth requires understanding the environmental dependencies and interactions between technological components.

The structure of this chapter is as follows. We develop our theoretical model and associated hypotheses in Section 2. After developing our theory, we will test a series of specific hypotheses that follow from this ecological logic through an empirical analysis of patents and patent citations in one of the leading sets of technology developed in the 20th century [9]: i.e., biotechnology.Note that given our focus on patent data, we operationalize growth as entry (normally, growth is equated with net entry–i.e., entry minus exit), as patents do not exit. Hence, in the remainder, technological growth and entry are used interchangeably. In Section 3, we elaborate on the empirical setting of our study, introduce our empirical measures, and explain our estimation methods. Section 4 presents the results of our empirical analyses. And finally, in Section 5, the findings are discussed in relation to our theory and the broader literature.

## The Technological Niche

The concept of the niche was first developed by [10], and is still central to many ecological studies today, where it is used to delineate the relational position of an organism, population or species in an ecosystem. Within the social sciences, the niche has received widespread attention in numerous empirical studies [7, 11, 12], as well as theoretical work [13, 14, 15]. The technological niche concept was first developed by [7] to investigate the effects of crowding and status on the future importance of individual inventions. They defined the technological niche as the relational context of an invention that co-evolves with technological change. In [12], the concept of the technological niche was subsequently applied at the organizational level of analysis, to study the effects of crowding and status on organizational growth and survival. In the current study, we want to continue in this tradition, and build on this notion of the technological niche.

We define the technological niche as the relational context of a technological component (e.g., genetic engineering), embedded within a technological system (e.g., biotechnology). Hence, we view technology as a system that cuts across organizational boundaries [16], and abstract from the individual organization, by focusing instead on the aggregate pattern of development of all organizations that are active in a certain technological system (i.e., biotechnology). Accordingly, we use the ecological framework of [17] to study technology in terms of its elemental components. So, in analogy with [18], we define a technological system as a bounded set of technological components with a related identity. In turn, a technological component is defined as a population of related technological inventions. In doing so, we develop a multi-level model of endogenous technological growth.

Our key dependent variable is growth of the technological component niche as reflected in entry by new inventions, coined component growth. Below, we will subsequently discuss the dimensions of the component niche central to our theory that we take as our independent variables, focusing on both a component niche’s internal (i.e., component and system density, and component diversity) and external (i.e., component and system crowding, and component status) features. Note that, given our application to component niches of biotechnology, we often refer to the short-cut component for component niche.

### Component density

Ecologists have observed a characteristic pattern of evolution of diverse organizational populations: initially, after a slow kick-off, population size–measured in terms of the number of organizations, or density–increases rapidly, and then stabilizes or even declines in numbers [19]. Intrigued by the universality of this typical S-shaped growth path (which has also been recorded in studies on technological innovation and diffusion, cf. [20, 21, 22]), organizational ecologists have sought to explain this phenomenon. They were able to do so by integrating elements from ecological and institutional theories, into what is now known as density dependence theory. This theory posits that the two general forces of selection–i.e., social legitimation and diffuse competition–are linked to the density of organizational populations. Basically, population density serves as a surrogate for the difficult-to-observe features of the material and social environment that affect organizational founding and mortality rates, particularly competition and legitimation [19].

Legitimation refers to “the standing as a taken-for-granted element in a social structure” [15], and is especially important in the early stages of population development. The capacity of an organizational form to mobilize resources is to a large extent dependent on the degree in which (extremely skeptical) resource controllers take the form for granted [23, 24]. Legitimation is tied to density because, according to [25], “if institutionalization means that certain forms assume a taken-for-granted character, then simple prevalence of the form ought to legitimate it.” Legitimation processes thus produce a positive relationship between population density and founding rates.

Density also has an obvious link with diffuse competition, which is defined as common dependence on the same resource pool. If density increases linearly, the number of potential competitive links increases exponentially [24]. This implies that density increases diffuse competition at an increasing rate, as more organizations fight for limited resources, resulting in declining founding rates and increasing mortality rates [25]. This is standard economics logic. The joint forces of legitimation (dominant at low density) and competition (dominant at high density) produce non-monotonic density-dependent processes of organizational entry (hill-shaped) and exit (U-shaped), which together generate an S-shaped growth curve of population density.

Even though the theory of density dependence has been primarily applied to organizational populations, and very successfully so, recent research illustrates that, due to its general nature, this argument can also be effectively applied in other settings, such as the birth and death rates of national laws [26, 27] and organizational rules [28, 29]. In a similar vein, we believe that density dependence logic can fruitfully be used in the study of evolutionary processes within technological populations, cf. [30]. However, we have to keep in mind that, even though similarities between technologies and organizations provide a useful platform for exporting analytical concepts from one domain to the other, we have to be careful not to equate one sphere with the other [30]. This implies that we should systematically consider the extent to which processes of competition and legitimation operate in technological populations, within and across niches.

It is widely acknowledged that technologies need to be legitimized [23, 31]. According to [32], organizations even adopt technology to enhance their own legitimacy. Hence, technologies are then institutionalized, becoming a taken-for-granted means to accomplish organizational ends [33]. This process of legitimation is especially important in the formative stage of a technology when, akin to the initial stages of organizational populations, “important constituents, such as investors, founders, potential customers and employees lack a clear understanding of the newly emerging activity, hampering taken-for-grantedness and resource mobilization” [34]. Here, we believe that the denser the component’s technology (i.e., the more technological inventions there are in the component’s niche), the better understood the technological component is, and the more it is taken-for-granted as the appropriate means to accomplish a certain goal (e.g., use rDNA technology to modify the genetic structure of living material). This process enhances the growth of this technological component. Analogous to the acceptance of a new organizational form by society, legitimacy of a new technological component increases with the number of technological inventions in the component’s niche. Hence, at low levels of component density, we expect that component density stimulates further component entry.

Ideas and innovations compete with one another for the attraction of resources and attention [7]. That is, due to the scarcity of stakeholder resources, only a limited amount of resources and attention can be attributed to (a particular kind of) technological development at a certain point in time. Because a firm’s research budget or an investor’s capital is limited, alternative inventions compete for these scarce resources. Increasing density increases the number of inventions that depend upon these scarce resources for further development (e.g., successful introduction into the market by turning the invention into an innovation). So, when these resources become scarce at high levels of component density, processes of competition start to develop between alternative inventions. Hence, at high levels of component density, component density hampers component entry.

**Hypothesis 1**: There is an inverted U-shaped effect of component density on component growth.

### System density

Over the years, density dependence theory has received considerable critique. This is mainly the result of the generality of the model. On the one hand, regarding the legitimation processes, opponents–mainly institutionalists–argue that legitimation is a multi-dimensional construct that cannot be adequately represented by a measure as crude as population density [31]. This critique argues that population evolution is highly dependent on idiosyncratic events (e.g., legislative changes, overt political support, and entrepreneurial initiatives) that are largely ignored when merely studying population numbers. Accordingly, ecologists argue that those events are indeed important, but can never be fully taken into account by any general theory, and therefore opt to control for such population-specific events instead. On the one hand, we follow the ecological approach in this matter by controlling for specific events (e.g., by including time-specific dummies). On the other hand, we take the institutional approach by introducing, in addition to population density, component status to represent the processes of legitimation (see below).

The competition leg of the theory has also been challenged. It is argued that populations are not fully homogeneous, and that segments of the population respond differently to (mainly) competitive processes [35]. Indeed, further research indicates that competitive processes are highly localized because competition is tied to material resources (i.e., plants, products, and people), and is therefore hampered by spatial and geographic boundaries [24, 35]. In contrast, legitimation processes are tied to information, which flows more freely, and is therefore hampered less by spatial boundaries. Accordingly, legitimation processes are argued to operate more broadly than competitive processes [24]. This provides fertile ground for extending the original density dependence model.

One of the proposed extensions is to employ multi-level models, where processes of legitimation are allowed to operate more broadly than competitive processes [36]. Here, we follow this line of reasoning by arguing that the flow of material resources (i.e., plants, products, and people) is not only disrupted by physical and political barriers [24], but also by technological boundaries. That is, we claim that technology also localizes competitive processes, whilst processes of legitimation operate on a broader technological scale. Hence, we expect density within the entire technological system to be tied to processes of legitimation (and not to competition). After all, a set of components comprise a system only when these components form an integrated whole–that is, when the whole is greater than the sum of its parts.

**Hypothesis 2**: System density is positively associated with component growth.

### Component diversity

Because different population segments may well respond differently to processes of competition and legitimation, it is important to consider whether a population is subdivided into segments. In the context of our current study, three motives come to mind for considering such across-niche diversity. First, according to [37], there is an inverse relationship between diversification (i.e., diversity) and competition. That is, if a population becomes more diverse, the level of competitive intensity within the population decreases. This is reflected in economics’ theory of product differentiation [38]. Therefore, as the rate of entry is tied to the competitive intensity within a technological component, we expect component growth to increase with component diversity.

Second, diversity mitigates lock-in and provides flexibility in uncertain environments [39]. Because technological development within biotechnology is of a highly uncertain nature in our time window, flexibility is important by providing alternative directions for future development. In this sense, diversity is indicative of niche width, and increasing the diversity of the niche increases the latter component’s potential applicability in the wider environment, implying that it is appealing to a greater variety of stakeholders, which positively affects the rate of component entry.

Third, diversity is one of the central principles in evolutionary economics, see [40, 41]. Just as an “economy with no diversity and no selection will not evolve” [42], a technology with no diversity and no selection will also not evolve, but will instead remain permanently locked in a steady state equilibrium. Technological change is a process of recombination, so increasing the number of subcomponents (in a component niche) increases the opportunities for their (re)combination, yielding further opportunities for the creation of new inventions.

**Hypothesis 3**: Component diversity is positively associated with component growth.

### Component status

Even though processes of legitimation at the system level affect all components within the system, it is highly unlikely that system-level legitimation will affect all components equally. Furthermore, according to the institutional argument, component density is a proxy that cannot accurately capture the distribution of system-level legitimation among components. This means that we need another way to distinguish between the legitimation of individual components relative to the other components within the technological system. A well-known construct that measures legitimation at the individual member level is status, which is defined as a focal member’s ‘perceived’ quality in relation to the ‘perceived’ quality of other population members [43, 44].

Status is an instance of endogenous system or population structuring that results from the interactions among members and stakeholders in a population. Akin to the importance of legitimation in the formative (or uncertain) stages of population development, status is mainly used by resource controllers to guide their decisions in uncertain environments. Due to the uncertainty, the quality of population members cannot be objectively determined. Therefore, resource controllers rely on status to guide their decisions [44]. In the context of technological development, the role of status has been studied by [7] and [12].

According to these studies, as the uncertain environment makes quality perceptions dependent on status, status becomes important in guiding the flow of resources in technological developments. As other organizations build upon the focal organization’s technology, a certain status is conferred to that focal organization’s technology [7]. Here, akin to the explanation at the organizational level, we argue that, when aggregate technological developments build upon a focal technological component, a certain status is transferred to the focal component as this provides a signal to the stakeholders of the technological system that the focal component is worthy of attention and resources. So, in times of uncertainty, high-status components offer an anchor for technological investment (i.e., resources), attracting component entry. In [12], the authors refrain from hypothesizing about the main effect of status because, as they argue, “one cannot specify an average status effect independent of a meaningful assessment of the average crowding or uncertainty in a technological domain.” However, because technological developments within biotechnology can be characterized by high levels of Knightian uncertainty [12, 45], we expect component status to have a positive main effect on component entry.

**Hypothesis 4**: Component status is positively associated with component growth.

### Component crowding

In ecological studies, niche crowding is usually equated with competition, as it implies a similarity in resource requirements [12, 15], which builds upon the notion that the potential for competition is directly proportional to the overlap of resource bases [11]. In view of our technological components, the resource base can be properly represented by the knowledge on which the inventions within the component build. Especially when markets are not (yet) existent, technological development is to a large extent dependent upon the underlying knowledge base [46].

Because inventions recombine technology from prior antecedent inventions, these prior or antecedent inventions constitute the building blocks of the focal inventions. The uniqueness of the invention’s building blocks determines the uniqueness of the invention itself [2]. Aggregated to the component level, this implies that the more a focal technological component builds upon unique elements, the more unique the focal component itself is. So, we define the technological antecedents of our focal technological components with reference to their knowledge or resource base. An overlap in technological antecedents increases the competition experienced by the component, because it decreases the uniqueness of the technological component. Consequently, we argue that competition not only occurs within a technological component (as argued under component density), but also between technological components.

However, niche crowding can also contribute in a positive way to niche entry or growth, due to knowledge and reputation spillovers [47, 48], economies of standardization through sharing of infrastructure [49], and vicarious learning [50]. This mutualistic relationship has been validated empirically in numerous studies, cf. [2, 48, 51, 52]. Here, we explore the extent to which these arguments can be applied as well when investigating aggregate patterns of technological development.

First, technology can become a taken-for-granted means to accomplish an organizational objective, implying the existence of legitimation or reputation spillovers from (the use of) one technology to the (use of the) other. Furthermore, knowledge spillovers within technological development are well-documented. As the usage of technology increases, the documentation of technology also increases–for example, in patent documents, manuals, and books. As a result, the characteristics and behaviors of often-used technologies are better known.

Second, economies of standardization or infrastructure sharing relate to the costs of transportation, communication, and ease of supply [49], which are important in the case of technological development, too. Consider, for example, the use of active compounds (e.g., molecules and proteins) in biological tests. Reliance on compounds that are not readily available obviously hampers technological development. Moreover, according to [30], an emerging technology can benefit from the infrastructure that was created to accommodate the mature technology. Third, and finally, vicarious learning is possible through adaptation and selection of ideas, structures, and technologies [50], which by definition play an important role in technological development.

How can we accommodate for both a positive and negative effect of component crowding? The answer to this question lies in an analysis of the spatial pattern of crowding. The investigation of spatial patterns in the distribution of organisms is central in ecology, as they play an important role in population dynamics [53]. According to [54], the “distribution of nutrients as well as interactions on spatial scale like migration can have important impact on dynamics of ecological populations.” Likewise, the investigation of spatial patterns in the distribution of organizations is central in organizational ecology. According to organizational ecology logic, processes of competition are more localized than processes of legitimation. More local or more similar organizations are more likely to vie for the same pool of resources. In a similar vein, we argue that more local technological components compete for the same resources, such as venture capital, investments, and research budgets. The competitive processes are bound by technological systems. This means that we distinguish between two forms of crowding. On the one hand, local crowding refers to crowding of our focal components amongst themselves: i.e., crowding within our technological system, reflecting the extent to which our focal biotechnology components build upon the same antecedent technology. On the other hand, global crowding refers to crowding of our focal component by non-focal components: i.e., crowding of our focal components by components outside the technological system, reflecting the extent to which biotechnology’s components build upon the same technology as non-biotechnology components.

**Hypothesis 5**: Local crowding is negatively associated with component growth.

**Hypothesis 6**: Global crowding is positively associated with component growth.

We have argued under component status that a certain amount of legitimacy is transferred to a focal organization when another organization builds upon the focal organization’s technology. However, according to [12], such a technological tie between two organizations also implies that their technologies are similar, which increases the potential for competition between the two organizations. They reason that these technological ties have the most potent competitive impact in crowded regions of the network, resulting in clique-like structures among structurally equivalent organizations. They therefore claim that the effect of status is positive in uncrowded niches, and that this positive effect decreases with niche crowding. Similarly, we expect that these technological ties can have competitive implications in our setting as well. However, because competitive processes are bound by technological systems, the effect of status only decreases with local crowding, and not with global crowding.

**Hypothesis 7a**: The interaction term of local crowding and component status is negatively associated with component growth.

**Hypothesis 7b**: The interaction term of global crowding and component status is not negatively associated with component growth.

## Methodology and Data

Patents and patent citations provide the core of the data that we will use to test our hypotheses. Patents and patent citations have been used extensively in the study of technological change and organizational innovation [2, 7, 47, 55]. Obviously, patent data do not capture the full spectrum of technological development, as not all technology is patented. Patents only represent the explicit part of technological development and do not capture the tacit part of technological development. While differences might exist in different industries in the ratio between tacit and explicit technological development, within the same industry this ratio will be relatively stable (depending upon the ease and strength of patent protections). As such, within industries, the explicit part of technological development can be used as a proxy for the whole of technological development. Especially within biotechnology, patents form a reliable indicator of technological developments [56, 57], as all landmark innovations have been patented.

Previous research has illustrated that the US patent system offers the most complete dataset for technological analysis, since the US is the world’s largest and most international marketplace [7]. Furthermore, because the US is a large and central market for biotechnology, it is standard practice of biotechnology companies from outside the US to patent in this country [58]. We therefore use patent data from the United States Patent and Trademark Office (USPTO) in our empirical analysis. Patents are classified by the USPTO following a hierarchical classification system, known as the United States Patent Classification System (USPC), which is divided into 375 main classes that jointly contain about 125,000 sub-classes. For a patent to be granted, the applicant must establish the novelty of the invention relative to all previous inventions. This novelty claim is established by identifying and citing what is referred to as “prior art”. These citations are usually supplemented during the review by the patent examiner [2]. Previous research has clearly demonstrated the importance of patent citations [2, 47, 59, 60]. We therefore use these citations to delineate technological lineage and the embeddedness of a focal technological component in the broader technological environment.

Biotechnology patents are registered in classes 435 and 800 of the USPC. The domain of biotechnology has an average of 57 per cent of self-citations, and can therefore be considered as highly autonomous and independent. Hence, biotechnology offers a setting highly suitable for an empirical investigation of the kind proposed here. The biotechnology domain contains 27 main sub-classes (i.e., 18 in class 435 and 9 in class 800), as listed in S1 Appendix. As argued, we define our technological niches (i.e., components) at this level of analysis.

### Measures

Component growth, our dependent variable, is measured by the count of the number of patents that enter our focal components in a particular month in the period between 1976 and 2003. As we have repeated observations for the same components, our data form a time-series–cross-sectional panel. This panel is unbalanced, though, as not all components were in existence at the start of our time window.

Focal Component density is a count of the total number of patents (divided by 1000) in the focal component in the month prior to the date of measurement of our dependent variable. So, this measure represents the stock of patents contained in the focal component. System density is a count of the total number of patents (divided by 1000) within the domain of biotechnology (i.e., USPTO classes 435 and 800) in the month prior to the date of measurement of our dependent variable. To avoid double counting, we subtract focal component density from system density.

Component crowding refers to the extent to which the building blocks (i.e., antecedent inventions) of our focal components are crowded (or used) by other components. To provide for a baseline model to test our hypotheses regarding the distinction between local and global crowding, we calculate the aggregate measure of crowding (i.e., Total crowding) as follows:

$$CO_{it}=\sum_{j=1;j\neq i}^{J}\sum_{k=1;k\neq i;k\neq j}^{K}\frac{\left|A_{ikt}\cap A_{jkt}\right|}{\left|A_{ikt}\right|},$$ (1)

where *CO*_*it*_ refers to the overlap experienced by focal component *i* at time *t*, *A*_*ikt*_ to the antecedent inventions of component *i*’s inventions from component *k* at time *t*, *A*_*jkt*_ to the antecedent inventions of component *j*’s inventions from component *k* at time *t*, *J* and *K* to the set of all components, ∩ to the intersection of two sets (i.e., the common elements in both sets), |.| to the cardinality of a set (i.e., the number of unique elements contained within the set), and *t* to the twelve months prior to the month of observation of our dependent variable. To make the number of non-focal components manageable, we have defined the non-focal components at the class level instead of the sub-class level. Strictly speaking, this implies that our non-focal components are actually composed of alternative technological systems.

**Fig 1:**
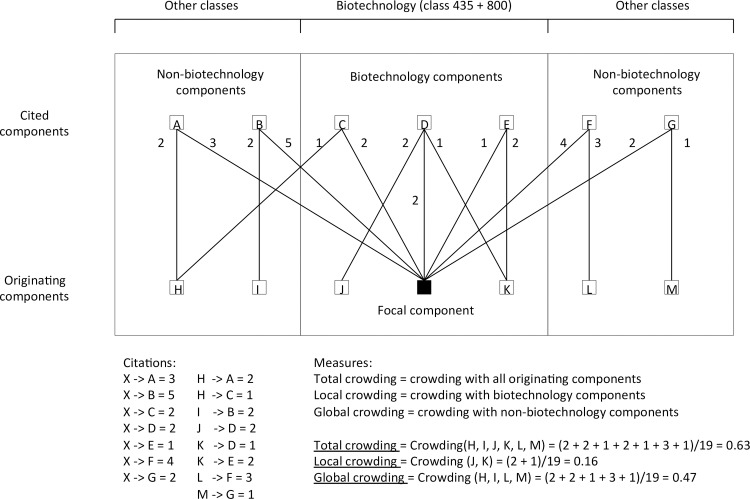
Example: calculation of total, local, and global crowding. Total crowding calculates the overlap in citations (of antecedent inventions) between our focal and all technological components. Local crowding calculates the overlap in citations between our focal and biotechnological (local) components only. Global crowding calculates the overlap in citations between our focal and all non-biotechnology (global) components.

For our measure of Local crowding, we calculate the overlap of our focal components using (1). In this case, *J* refers to the set of focal components only, while *K* denotes the set of both focal and non-focal components. Global crowding measures the overlap of our focal components with the non-focal technological components. To calculate this measure, we again use (1), but now *J* refers to the set of all non-focal components, whilst *K* is the set of all components (so both focal and non-focal). An example of how our crowding measures are calculated is provided in Fig 1.

Focal Component status is based upon the patent citations received by the focal component. Patent citations reveal system-wide perceptions of the relative importance of patented technologies [60, 61], and can therefore be used to measure the status of the component. In line with [7], we use a ratio for component status to correct for the expanding risk set of patents in our components. The number of patent citations that a component receives is to a large extent dependent upon the number of inventions that are contained within the component (i.e., component density). Therefore, we divide the number of patent citations by the density of the component. This significantly reduces the correlation between, on the one hand, component status, and, on the other hand, component density and organizational density. This implies

Sit=∑j=1JCRijtDit, (2)

where *S*_*it*_ is the status of component *i* at time *t*, *CR*_*ijt*_ denotes the number of citations received by invention *j* in focal component *i* at time *t*, *J* refers to the set of all inventions in component *i*, *D*_*it*_ indicates the density of component *i* at time *t*, and *t* captures the twelve months prior to the month of observation of our dependent variable. We exclude self-citations as these do not adequately reflect the public deference process that this variable is supposed to represent [12].

Focal Component diversity is measured via the distribution of patents across sub-components or population segments contained in the focal component over the previous twelve months. These sub-components are represented by the USPC sub-classes that are associated with the focal component. To measure component diversity, we use the diversity measure from [62]:

$$CD_{it}=\sum_{j=1}^{j=J}P_{ijt}\ln(1/P_{ijt}),$$ (3)

where *CD*_*it*_ refers to the diversity of component *i* at time *t*, and *P*_*ijt*_ is the share of patents in sub-component *j* of component *I* at time *t*, *J* denotes the total number of sub-components of component *i*, and *t* captures the twelve months prior to the month of observation of our dependent variable.

Our first control variable is Organizational density, which is a count of the number of organizations (in thousands) active in the technological component in the twelve months prior to the month of observation of our dependent variable. We expect a positive effect of organizational density on component growth, as the legitimation of technology is to a large extent determined by the number of organizations that adopt the technology [46]. Initially, increasing the number of organizations increases the rate of scientific discovery. However, at some point, increasing the number of organizations implies that the chances for discovery decrease. Under these circumstances, the best defense or strategy for organizations is to control as many pieces of technology as they can [52], as these can be used as leverage (i.e., bargaining power) in the competitive arena. This leads to ineffective strategies of technological development, depressing the technology’s growth. Hence, we expect to find a hill-shaped effect of organizational density on component growth.

We also include Year-month dummies in all our analyses to control for year-month specific effects. Furthermore, in accordance with prior research, we also add the number of previous entries and its square–Previous entry and Previous entry2 –to control for favorable conditions within the environment that may encourage niche entry [36]. Table 1 gives the summary statistics, and Table 2 presents the correlation matrix. Organizational density, component density, and previous entries reveal high multicollinearity, which means we have to proceed with some caution.

**Table 1:** Descriptive statistics.

Variable	Obs.	Mean	Std.dev.	Min.	Max.	Q_1_	Q_2_	Q_3_
Component growth	8,021	5.02	14.35	0	217	0	1	4
Previous entries	8,021	0.00	0.01	0	0.22	0.00	0.001	0.004
Organizational density	8,021	0.03	0.08	0	0.67	0.00	0.01	0.03
Component density	8,021	0.67	1.63	0.00	15.14	0.02	0.09	0.57
System density	8,021	16.55	11.17	2.88	44.95	7.70	12.55	22.61
Diversity	8,021	1.83	1.50	0	4.71	0.00	1.93	3.17
Component status	8,021	0.33	0.32	0	3.00	0.12	0.26	0.45
Total crowding	8,021	0.08	0.06	0	0.31	0.03	0.08	0.11
Local crowding	8,021	0.09	0.08	0	0.38	0.03	0.08	0.14
Global crowding	8,021	0.68	0.53	0	2.71	0.22	0.69	1.00

Q1, Q2 and Q3 refer to the 25th, 50th and 75th percentile, respectively.

**Table 2:** Correlation matrix.

Variable	1	2	3	4	5	6	7	8	9
1. Component growth									
2. Previous entries	0.93*								
3. Organizational density	0.94*	0.94*							
4. Component density	0.88*	0.88*	0.95*						
5. Community density	0.11*	0.12*	0.14*	0.10*					
6. Diversity	0.38*	0.38*	0.46*	0.48*	-0.08*				
7. Component status	0.00*	0.00	0.00	-0.04*	0.43*	-0.14*			
8. Total crowding	-0.11*	-0.11*	-0.10*	-0.12*	0.25*	0.10*	0.20*		
9. Local crowding	-0.08*	-0.08*	-0.07*	-0.11*	0.56*	0.00	0.37*	0.82*	
10. Global crowding	-0.11*	-0.11*	-0.11*	-0.12*	0.20*	0.11*	0.17*	1.00*	0.77*

* p < 0.01.

### Data description

**Fig 2:**
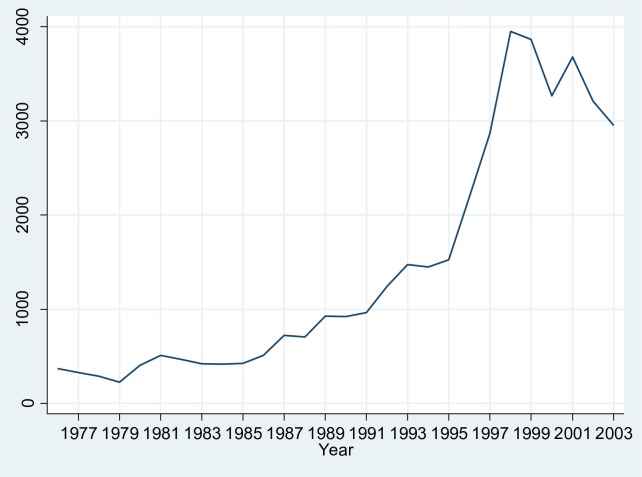
Yearly entry rate of biotechnology patents. The yearly entry rate of biotechnology patents shows a steady increase until 1998, after which we can witness a decline in the number of patents. This seems to indicate that biotechnology has entered into a different stage of technological development, in accordance with the S-shaped growth path commonly reported in technological development.

If we take a closer look at the data regarding the individual components, as summarized in Table 3, we can make the following observations. First of all, biotechnology is dominated by a small number of components. The five largest components (i.e., subclasses 435004, 435005, 435009, 435011, and 435014) jointly account for more than 75 per cent of all biotechnology patents. Second, as can be seen in Fig 2, the growth in biotechnology patents peaked in 1998 (with 3,952 patents), after which we can witness a decline in the number of patents per year: There seems to be a leveling-off in the growth of biotechnology patents in the post-1998 period.

**Table 3:** Descriptive statistics of individual component niches.

		Entry		Density	Status	Crowding	Local	Global	Diversity	Density	
Niche	Obs.	Mean	S.D.	Mean	Mean	Mean	Mean	Mean	Mean	share	Group
435001	336	0.61	0.93	72	0.60	0.10	0.11	0.90	1.08	0.49%	3
435002	336	0.86	1.14	133	0.41	0.11	0.11	0.94	0.00	0.73%	1
435003	336	0.14	0.36	51	0.31	0.10	0.10	0.95	0.00	0.16%	4
435004	336	44.15	43.60	4,379	0.26	0.04	0.03	0.37	3.58	33.89%	2
435005	336	26.58	21.00	4,946	0.16	0.05	0.04	0.42	4.57	24.43%	1
435006	336	2.49	3.50	174	0.39	0.10	0.13	0.91	3.00	1.84%	2
435007	336	0.41	0.74	60	0.34	0.10	0.10	0.93	1.83	0.36%	2
435008	336	1.71	1.43	380	0.49	0.11	0.12	1.00	2.05	1.48%	4
435009	336	9.08	8.43	1,577	0.14	0.08	0.09	0.70	3.64	8.22%	1
435010	336	1.31	1.71	173	0.32	0.09	0.15	0.77	1.36	1.08%	1
435011	336	6.68	8.23	507	0.36	0.09	0.10	0.80	3.61	5.01%	3
435012	336	0.87	1.21	84	0.33	0.08	0.10	0.66	1.96	0.65%	1
435013	336	0.04	0.22	26	0.07	0.02	0.03	0.17	0.00	0.07%	6
435014	336	5.74	4.51	1,106	0.17	0.08	0.09	0.68	3.20	5.31%	3
435015	243	3.00	3.41	201	0.45	0.11	0.17	0.97	0.00	1.62%	2
435016	336	2.74	2.73	713	0.15	0.10	0.11	0.85	2.83	2.99%	1
435017	336	5.20	4.19	1,047	0.25	0.08	0.05	0.75	4.15	4.86%	2
435018	336	0.04	0.21	11	0.27	0.05	0.06	0.40	0.00	0.04%	6
800001	121	0.66	0.89	32	0.23	0.14	0.20	1.20	0.00	0.18%	5
800002	159	0.24	0.52	13	0.60	0.11	0.16	0.91	0.76	0.08%	5
800003	177	1.53	1.92	69	0.67	0.09	0.15	0.73	1.31	0.60%	2
800004	203	0.32	0.63	18	0.70	0.08	0.12	0.70	1.14	0.15%	2
800005	336	1.27	2.76	127	0.34	0.07	0.09	0.62	2.09	0.97%	4
800006	225	0.06	0.23	8	0.71	0.02	0.05	0.15	0.00	0.03%	6
800007	336	0.02	0.16	4	0.22	0.01	0.01	0.06	0.00	0.02%	6
800008	200	4.11	4.61	206	0.26	0.09	0.13	0.75	2.08	1.82%	2
800009	336	3.94	7.03	194	0.30	0.04	0.05	0.38	1.79	2.90%	1

**Fig 3:**
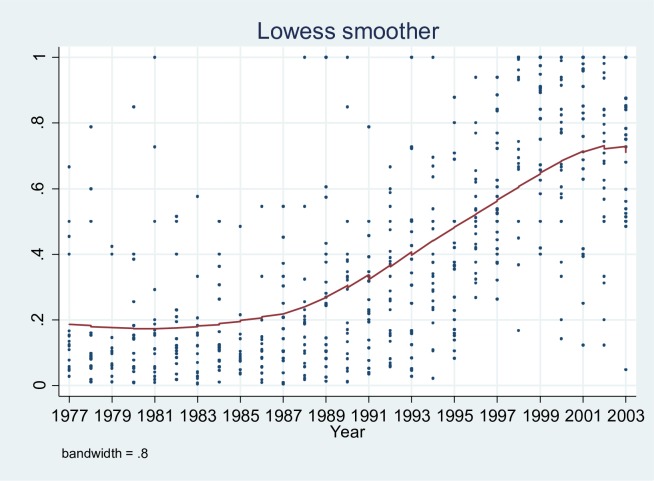
Locally weighted regression (lowess) of relative entry per niche. While a clear decrease in the ‘average’ relative entry of patents in component niches cannot be observed in this figure, a decreasing growth rate in the relative entry rates (or leveling-off) can clearly be established, again in accordance with the S-shaped growth path of technological development.

However, because the absolute number of patents in biotechnology is clearly dominated by the largest components, we need to look at the individual niches to determine whether this leveling-off is actually a pattern that can be observed for all niches. To do so, we ran a locally weighted regression (lowess) on the basis of the relative entry (the relative entry of a component is defined as the number of patents entering the component in a year divided by the maximum number of patents that enters the components in any given year) in the individual niches for the different years, as visualized in Fig 3. The outcome seems to confirm the leveling-off for the majority of biotechnology’s components.

**Fig 4:**
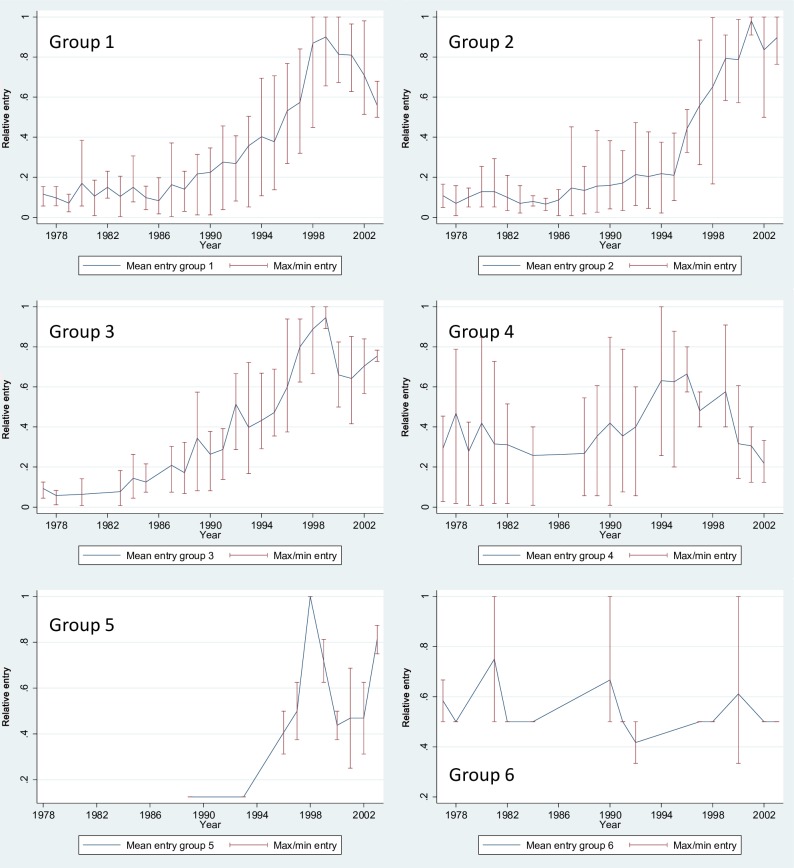
Yearly entry in clustered component niches. As we can learn from Table 3, the major components are in groups 1 to 3, which display a common pattern. Groups 1 to 3 all peak between 1998 and 2000, and the main different between these groups of components is what occurs after this peak. The growth of the components in group 1 declines, whereas the growth of components in groups 2 and 3 first declines but then picks up again. Groups 4 to 6 show a rather erratic entry rate. The reason for the difference in growth patterns of individual components is that they are located differently along the technological lifecycle. Some are in the beginning of their development while others seem to be more fully developed.

As can be seen by the scattering of points in Fig 3, there is a great variation in the rate of growth across the individual components. Therefore, we have grouped the individual components on the basis of their entry patterns. As Fig 4 reveals, this gives six groups: (1) 435002, 435005, 435009, 435010, 435012, 435016, and 800009; (2) 435004, 435006, 435007, 435015, 435017, 800003, 800004, and 800008; (3) 435001, 435011, and 435014; (4) 435003, 435008, and 800005; (5) 800001 and 800002; and (6) 435013, 435018, 800006, and 800007.

On the basis of this descriptive data analysis, we draw two main conclusions. First, there seems to be a leveling-off in biotechnology’s major components, which suggests that these components have entered into a different stage of evolutionary development. Second, there is a difference in the evolution of the growth of individual biotechnology components, which warrants an analysis at the component level, as we will conduct below.

### Estimation

In ecological studies, the number of entrants is a natural and intuitive dependent variable. In organizational ecology, indeed, organizational founding studies abound. Similarly, the entry of inventions or patents in our technological components can be considered as an arrival process. Arrival processes count the number of arrivals to some state. The natural baseline model for arrival processes is the Poisson specification [19], and adding the effects of covariates gives the Poisson regression model. However, using the Poisson distribution for modeling economic events involves quite strong and empirically questionable assumptions [63]. Empirical research on patent rates rarely finds that the mean of a time series of arrivals equals the variance, as a Poisson process implies. Instead, the variance tends to exceed the mean. This gives so-called overdispersion. The sources of overdispersion include, for instance, unobserved heterogeneity and time dependence [24]. Analysis of our sample clearly demonstrates overdispersion (if the sample variance is more than twice the sample mean, the data most likely suffer from overdispersion [63]). One way to deal with overdispersion is to allow for inter-component heterogeneity by permitting component *i*’s arrival rate *λ*_*i*_ to vary randomly according to some probability law. When f(*λ*_*i*_) is assumed to be a gamma distribution, we have a negative binomial specification [63]. Since the negative binomial specification allows for an additional source of variation, the estimated standard errors are larger, and the conclusions drawn are hence less precise [64].

Our data reflect an N-T panel structure, observing a cross-section of N niches over T months. Panel models accommodate for serial correlation (i.e., unobserved heterogeneity) between the repeated observations of the observed entities [64]–technological components, in our case. A negative binomial panel model can be represented by [65]:

$$\log\lambda_{it}=\beta X_{it}+\gamma \varepsilon_{i}+\mu_{i},$$ (4)

where X_*it*_ is a vector of characteristics of component *i* at time *t*, *γ* is a correction for overdispersion, and *μ*_*i*_ is a time-invariant effect for each entity or component *i*, reflecting micro-level heterogeneity. This parameter can be treated as either fixed or random.

Our data involve left-censoring, as information is missing for the beginning of the history of the population–that is, biotechnology. Patent citation data are not available for the pre-1975 period. This does not imply a survivor bias, though, as we do have all cohorts: none are missing. However, this could still distort our results because we do not have the full lifespan of our technologies. We do not think this poses a threat to our analyses, as the majority of developments within biotechnology have taken place after the discovery of recombinant DNA in 1972. Moreover, due to changes in patent law (i.e., the so-called Bayh Doyle Act, which allows the patenting of research findings funded by means of federal grants), commercial activity within biotechnology only took off after 1980 [55]; see Fig 3. Furthermore, we have data on a cross-section of different technologies within biotechnology, implying that several new and emerging technologies are represented. Nonetheless, we should still treat our findings with some caution [24]. Processes of legitimation are especially important in the formative stages of population development. So, left-censoring might result in finding competitive effects only, due to an under-representation of processes of legitimation. Hence, this implies that we should be especially wary if we find no evidence for legitimation processes. To determine whether or not progressive model extensions imply a significant improvement in model fit, we follow standard practice and compare twice the difference in the Log-likelihood to a χ^2^ distribution with degrees of freedom equal to the number of added variables. In doing so, stability of coefficient values over alternative models increases confidence in our findings.

## Results and Discussion

Table 4 presents estimates for our random effects negative binomial dispersion models of patent counts (all models were estimated with the ‘xtnbreg, re’ command in Stata 13). The parameter estimates are not standardized, which means that the coefficient values should be exponentiated before interpretation. The exponentiated coefficients represent multiplier effects on the rate of component growth.

**Table 4:** Negative binomial random effects panel regression estimates.

Variable	1		2		3		4		5		6		7		8		9	
Previous entries/1000	5.49	***	4.25	***	6.44	***	6.61	***	6.56	***	6.49	***	6.27	***	6.55	***	6.40	***
(Previous entries/1000)^2	-12.47	***	-9.60	**	-18.03	***	-18.51	***	-18.17	***	-17.87	***	-17.08	***	-18.09	***	-17.44	***
LN(Organizational density)	0.92	***	0.90	***	0.64	***	0.63	***	0.62	***	0.61	***	0.60	***	0.61	***	0.60	***
(Organizational density/1000)^2	-0.59	***	-0.85	***	-0.14		-0.05		-0.11		-0.06		-0.11		-0.09		-0.22	
System density/10.000			-8.51	***	5.25	*	3.75		4.21		4.72		5.14	*	4.54		4.19	
(System density/10.000)^2			0.09	***	-0.05	*	-0.04		-0.03		-0.03		-0.03		-0.02		0.00	
LN(Component density)					0.34	***	0.31	***	0.31	***	0.32	***	0.32	***	0.32	***	0.33	***
(Component density/10.000)^2					-0.50		-0.19		0.52		0.57		0.83		0.72		1.32	
Diversity							0.13	***	0.12	***	0.13	***	0.13	***	0.13	***	0.13	***
Component status (CS)									-0.20	***	-0.19	**	-0.18	**	-0.01		0.15	
Total crowding (TC)											-0.53				0.16			
Local crowding (LC)													-0.73	*			0.76	
Global crowding (GC)													0.01				-0.09	
Interaction CS * TC															-1.56			
Interaction CS * LC																	-3.08	***
Interaction CS * GC																	0.20	
Constant	-0.53	***	1.51	*	-2.94	***	-2.69	***	-2.89	***	-3.00	***	-3.19	***	-3.14	***	-3.43	***
r	30.95		29.75		40.48		47.49		45.99		42.58		40.37		43.41		40.47	
s	7.40		7.14		9.79		11.84		11.17		10.34		9.78		10.53		9.80	
Log likelihood	-11,025		-11,016		-10,945		-10,939		-10,936		-10,935		-10,933		-10,933		-10,928	

* p < 0.10

** p < 0.05

*** p < 0.01.

Model 1 is our baseline model, including only previous entries and organizational density. Model 2 adds system density, and comparing the Log-likelihood value of Model 1 (-11,025) with that of Model 2 (-11,016) shows that Model 2 significantly improves model fit, as with a χ^2^ of 18 (i.e., two times the difference in Log-likelihood) and two degrees of freedom, p is smaller than 0.01. Adding component density in Model 3 also significantly improves model fit (i.e., a χ^2^ value of 142 with two degree of freedom implies that p < 0.01). Next, Model 4 adds diversity, which again significantly increases model fit (i.e., a χ^2^ value of 12; p < 0.01), and adding component status in Model 5 also improves model fit (i.e., a χ^2^ value of 6 with 1 d.f.; p < 0.05). While including component crowding in Model 6 does not lead to an improved model fit (i.e., χ^2^ value of 2 with 2 d.f. implies that p > 0.1), adding both local and global crowding in Model 7 does improve model fit (i.e., comparing model Model 7 with Model 5 implies a χ^2^ value of 6 with 2 d.f.; p < 0.05). Model 8 includes the interaction between status and total crowding, and is a significant improvement vis-à-vis Model 5 (i.e., a χ^2^ of 6 with 2 d.f. implies that p < 0.05). Finally, Model 9 adds the interaction between, on the one hand, component status and, on the other hand, local and global crowding. This results in a significant improvement of model fit (i.e., compared to Model 8 this results in a χ^2^ of 10 with 2 d.f., so that p < 0.01). We use Model 9 to discuss the findings.

Hypothesis 1 states that there is an inverted U-shaped effect of component density on component growth, due to the twin effects of competition and legitimation. On the one hand, competition between ideas and innovations for the attraction of resources and attention results in a negative link between component density and component growth. On the other hand, especially in early stages of population development, legitimation also plays an important role. Legitimation results in a positive link between component density and growth, because increasing prevalence of a technological form increases its taken-for-granted character. Hypothesis 1 is partially supported because we only find a significant positive association between component density and component entry. Increasing density from its first quartile to its median value increases component entry with 65 per cent. Further increasing component density from its median value to its third quartile increases the rate of component entry with as much as 85 per cent. While we find a (non-significant) negative effect of the square of component density in Models 3 and 4, there is a switch in sign in Models 5 to 9 when we include component status and component crowding to our model. It thus seems that biotechnological components are mainly in the early stages of population development, where processes of legitimation dominate and competition processes are largely absent. In this stage, the competition processes that do exist are not well captured by the rather crude measure of component density, and component status and crowding appear to be better suited to measure these processes. Even though we do seem to witness a leveling off several biotechnological components in the years after 1999 (see Fig 4), our analysis comprises the period 1976 to 2003. Because the specification of our (estimation) model assumes a stable technological system, this leveling off does not become apparent in our results. Examining this requires a more dynamic model specification.

Hypothesis 2 states that system density is positively tied to component growth, due to positive spillovers between related (bio-)technological components or sub-forms. Hypothesis 2 is rejected, as we do not find a significant positive effect for system density. Even though we do find a positive effect of system density in several of our models, this effect is largely not significant. This points to the early stage of population development for biotechnology as a whole: few positive spillovers exist between technological components. This connects to the literature on dominant designs [66, 67, 68], originally conceived at the product level, but later found to be more relevant at a component level [69]. According to this literature, on the one hand, before a dominant design exists, actors recombine components and interact socially in an effort to find or become part of the dominant configuration that will serve as the basis for the future development of biotechnology. On the other hand, after a dominant design emerges, actors no longer invest in alternative configurations, but rather focus their attention on working out the component configuration represented in a dominant design, resulting in significant positive spillovers between individual components. Hence, over our period of observation, due to the absence of a dominant design, biotechnical development was mainly concentrated in individual components.

Hypothesis 3 argues that there is a positive link between component diversity and component growth, due to decreased competition, increased flexibility and an increase in combinatorial opportunities. This hypothesis is fully supported. We find a consistent and highly significant positive effect for component diversity. Regarding the effect of component diversity, increasing diversity with one standard deviation increases component growth with 22 per cent. The fact that diversity is significantly positive in all our model specifications points to the importance of including this measure in ecological models. However, given that biotechnology’s components are in the early stages of population development, it is still unclear whether the effect of diversity will remain positive in later stages of a component’s technological development.

In Hypothesis 4, we claim that component status is positively tied to component growth as it provides a signal to stakeholders that the component is worthy of resources and attention. We do not find support for this hypothesis: we even find a significant negative effect of component status in Models 5 to 8. Given that biotechnology is in its early stages of development and is therefore surrounded by great deals of uncertainty, this observation is quite remarkable. This seems to suggest that our current operationalization of component status does not seem to accurately capture the process of legitimation at the component level. Perhaps the number of organizations supporting technological developments in the component is better suited for this purpose. While we included the number of organizations primarily as a control variable, its strong positive and significant effect warrants for a more prominent role in an ecological theory of technological development and growth.

We do find full support for Hypothesis 5, which proposes that local crowding is negatively associated with component growth because competitive processes are more localized. As can be seen in Models 6 and 7, crowding does not have a significant effect on component growth until separated into its local and global representation. The coefficient for local crowding is highly significant and negative. In Model 7, increasing local crowding with one standard deviation decreases the rate of component entry with 6 per cent. In contrast to Hypothesis 5, we do not find support for Hypothesis 6. Hypothesis 6 suggests a positive link between global crowding and component growth, which results from positive spillovers, legitimation effects and economies of standardization. Even though we do find a positive coefficient for this measure, it is not significant. It therefore seems that total crowding is too crude a measure to accurately capture the process of competition in technological development. Due to the local nature of competition, attention needs to be paid to its spatial pattern.

Finally, Hypothesis 7a is fully supported by our estimates, while Hypothesis 7b is rejected. This pair of hypotheses is build upon the premise of localized competition and proposes a negative (positive) link between component growth and the interaction of local (global) crowding and component status. There is a highly significant negative coefficient for the interaction term between component status and local crowding (Hypothesis 7a), and no significant effect of the interaction term between component status and global crowding (Hypothesis 7b). However, the interaction term for status and global crowding is positive and could play a more important role in later stages of technological development.

Regarding the results for our control variables, we want to note the following. To control for year-month specific effects, we have included year-month dummies in our analysis (not reported here, for the sake of brevity; available upon request). No clear trend can be discerned. Although many individual year-months have a significant effect on niche growth, a clear evolution in either way cannot be observed. Next, with respect to the effect of previous entry, the coefficient for the linear term is positive and highly significant while the coefficient for the squared term is negative and highly significant, indicating a curvilinear effect of previous entry on subsequent entry. Adding one standard deviation from its mean value increases component growth with 9 per cent, while subtracting one standard deviation from its mean value also decreases component growth with 9 per cent. Finally, as already mentioned, organizational density has a highly significant effect on component growth. Increasing organizational density from its first quartile to its median value increases the rate of component entry with a staggering 295 per cent, while increasing the number of organizations from its median value to its third quartile further increases component entry with another 93 per cent. As said, this warrants a more central role for organizational density in a population ecology of technology.

### Robustness checks

We perform three robustness checks to make sure that our estimates do not suffer from misspecifications, unobserved heterogeneity, or other biases (all available upon request). First, to explore whether or not our findings are affected by multicollinearity, we estimated alternative specifications to represent the density-dependent processes, and build up our baseline model incrementally using stepwise regression. In the organizational ecology literature, two models can be found to test the density dependence argument. The original model is the Generalized-Yule (GY) specification, and the alternative is the Log-Quadratic (LQ) model. The GY model assumes a decreasing positive effect only, while the LQ specification also allows for an increasing positive effect. According to density dependence theory, each additional entry contributes less to the legitimation of the population. Therefore, in [70], the authors prefer the GY to the LQ model, the former connecting better to the original theory. However, they argue that when GY specifications do not converge or when LQ models result in a much better fit, LQ models can also be used. We estimated both the GY and LQ model for all our density measures (i.e., organizational density, system density, and component density), and have selected the specification that provides for the better fit. The results of the alternative specifications are highly similar: We have proceeded with the GY specification for both organizational and component density, and the LQ specification for system density.

Second, we examine the fixed versus random effects model. In our context, a complication is that the conditional fixed effects negative binomial model, as conceived by [64], is not a true fixed effects specification [71, 72], being based on a regression decomposition of the overdispersion parameter, rather than the usual regression decomposition of the mean [71]. As a result, the model only removes individual fixed effects equal to the logarithm of the overdispersion parameter [72], implying that the conditional fixed effects specification does not control for all stable covariates. Hence, there is no guarantee that the conditional fixed effects negative binomial model is completely free from serial correlation. As a consequence, the conditional fixed effects model does not provide for the consistent estimator required for the specification test in [73]. According to [71], a good alternative is to specify a conventional non-panel negative binomial model–NB2 in [63]–and manually add dummy variables to control for the fixed effects, which can be referred to as the unconditional fixed effects negative binomial model. However, because now T is fixed and N goes to infinity, the number of parameters increases with the number of cross-sectional observations, which results in biased coefficient estimates [74] by rendering the maximum likelihood estimator inconsistent [75]. This is referred to as the incidental parameters problem. However, despite an irrefutable theoretical inconsistency [76], [71] find no evidence for any incidental parameters bias for the unconditional fixed effects model in a simulation study of 100 panels and two time periods (i.e., N = 100 and T = 2). Our sample contains 27 technological components, and between 121 and 336 months (i.e., N = 27 and T > 120). Given the large number of time periods (T), we do not expect our data to suffer from the incidental parameters problem. We estimated both conditional and unconditional fixed effects specifications, as well as the random effects model. The conditional fixed effects model does not converge. Regarding the two remaining models, according to the specification test in [73], there is no significant difference between the coefficient estimates.

Third, we investigated the dynamic vis-à-vis dynamic panel estimation issue. Because we include a lag of the dependent variable (i.e., previous entry, and its square, into the component niche) in Table 3‘s equations, we are effectively estimating a dynamic panel model. According to [77], this might introduce a bias, especially when the number of time periods (i.e., T) is small. However, due to the large number of time periods in our data (between 120 and 335), we do not expect this to distort our findings. However, as a robustness check, we also estimated our final model without this lagged dependent variable. The pattern of findings of this static panel is highly consistent with our original random effects dynamic panel specification and does not lead to any change in our substantive arguments.

## Conclusion

On the basis of our results, we can conclude that during our period of observation, biotechnology’s components during our period of observation. In this stage, legitimation processes dominate competitive processes within technological components. As a result, we only find a significant positive effect of component density on component growth. Furthermore, a dominant design or technological paradigm appears to be absent at the system level (biotechnology as a whole) at this stage, as we do not find any significant positive spillovers between components at the system level. Diversity plays a key role in the early stages of technological development within components. Studying the role of diversity more directly in the evolution of technologies could thus lead to a better understanding of processes of endogenous technological change. In developing such a theory of diversity dependence in technological populations, both centripetal and centrifugal forces would have to be taken on board [17]. That is, we need a theory explaining when diversity stimulates or dampens technological growth (cf. [51]). Components status does not seem to be tied to the number of patent citations received, and organizational density seems to be a better proxy in the uncertain formative stage of technological development. The correct specification of competitive processes between components is highly important in this stage, as witnessed by our findings of the effects of local and global crowding and the negative effect of the interaction of local crowding and status on component growth.

The question of how technological changes come about endogenously has been left largely unanswered. One of the main reasons for this blind spot is that most studies view technology as a single component, without considering its multi-level nature. That is, these studies ignore the embeddedness of this component within a larger technological system, and thereby disregard the interdependence between components [78]. Hence, a systemic or structural perspective is relatively underdeveloped. The current study has addressed this gap, both theoretically and empirically, by developing and testing what we coin the ‘population ecology of technology’. The pattern of significant findings provides clear evidence for an ecologic dynamic of technological components within a technological system. Many ecological variables significantly impact upon a focal component’s growth. In all, we find full support for four hypotheses, and partial support for one. So, we believe that the ‘population ecology of technology’ proposed here is certainly promising, by applying ecological logic at the level of a technological system. Of course, our study is only a first step. Unexpected findings and design limitations offer steppingstones for future work. Here, we would like to reflect on five of these.

First, in developing a systemic view on technological growth, we have assumed that our technological system is stable, with technological components behaving in reliable and predictable ways. In doing so, we have been able to demonstrate that biotechnology–or any other technology, for that matter–can effectively be studied as a technological system, composed of a set of interdependent and interacting technological components. However, by no means does this imply that we perceive technology as a stable system, with components behaving in reliable and predictable ways. Even though we acknowledge that some technologies could, at a certain point in time, be characterized by such a stable state, at this moment, biotechnology is most definitely not one of them. On the contrary, biotechnology can be characterized as a highly dynamic technology, with many components that are just being developed [45]. Obviously, this implies that the patterns of interactions between biotechnology’s components have not yet stabilized. So, our current model is merely a steppingstone for the analysis of technology as a set of interdependent components. Our model needs to be extended to investigate the dynamics between these components over time. This could enable a distinction between the system’s core and peripheral components [69]. In turn, this would allow for studying the evolution of a technological system, driven by the evolution of its core components [79].

Second, an important limitation of our study is that we have abstracted from the role of the innovating organization. Our results clearly indicate that organizations play a major role in the growth process of a technological component. This signifies the importance of developing a co-evolutionary model, where both the evolution of technologies and the evolution of organizations are considered in unison. It is well-recognized that technologies and organizations/industries co-evolve [78, 80, 81]. We would like to briefly reflect on two obvious contributions that could be made when developing such a model. For one, it could lead to a theory that explicates the role of different organizational forms in a model of endogenous technological change. Technological change plays a key role in the creation of new organizations, and especially in the creation of new forms of organizations (i.e., form emergence). Each wave of technological change produces new sets of opportunities. While sometimes these opportunities are exploited by members of existing organizational forms, quite often only new organizational forms can effectively meet the requirements that arise from the application of new technology [19].

Moreover, at the level of an individual organization, we can relate the dynamics of technological components to the characteristics of an organization’s technological search behavior. Organizations search as members of a population [7], and by focusing on technology we basically investigate the search pattern of a population of organizations (i.e., a technological community or industry). By relating an individual organization’s technological search to the pattern of search at the organizational population level, it is possible to determine whether the organization’s search behavior conforms to or conflicts with this aggregate search pattern. This links to work done by [2], reporting an increase in the level of uncertainty and potential payoff of individual inventions when these inventions use more novel combinations. Moreover, this also connects to the notions of exploration and exploitation [82], and enables a distinction between processes of exploration and exploitation at the organizational level and the level of an organizational population (i.e., industry or community).

Third, another interesting avenue to consider are ecological models with spatial effects (cf. [54]). In our current investigation, we have made a distinction between global and local crowding to capture the spatial effects of crowding on component growth. Instead of a dichotomous distinction, we could develop continuous measures to more fully explore the spatial effects of crowding. Another option would be to investigate the role of migration (directed movement) and diffusion (random movement) in technological systems. Traditional ecological work generally uses reaction-diffusion models, on the basis of the assumption that population motion is random and isotropic [54]. Technological development is generally considered to be of a rational nature and migration models are more the norm. However, given the notion of bounded rationality, individuals and organizations do not have the ability to search the complete technological space. Therefore, technological development will also have a random component. Distinguishing between directed and random technological development could provide important insights into the evolution of technology.

Fourth, another limitation is that our empirical setting is the domain of biotechnology. Studying this technological domain has the advantage that patents form a reliable indicator of processes of technological growth [56, 57], hereby enhancing the internal validity of our study. However, a study into a single domain generally puts limits on the extent to which our findings can be generalized. Biotechnology reflects a highly science-based innovation pattern, with an important role for universities and research institutes. This clearly differs from technologies that are developed through inter-firm interaction, such as (lead) users and (specialized) suppliers [83]. So, different technologies are embedded in different patterns of interaction, which has consequences for the process of recombination. Studying such differential effects should be high on the agenda of future research in the realm of the ‘population ecology of technology’.

Fifth and finally, in this paper, we have applied insights from organizational ecology to develop a model of endogenous technological growth. This demonstrates how organizational ecology can contribute to the field of evolutionary economics [84]. This is not surprising, given that organization ecology and evolutionary economics are highly complementary fields. Both have their roots in biology, and build upon a model of variation, selection and retention, albeit with a slightly different focus. While organizational ecology places more emphasis on the process of selection, evolutionary economics underlines more the process of variation (cf. [85, 86, 87, 88, 89, 90]). Combining insights from both disciplines into a single model of variation, selection, and retention can contribute to the development of a more-encompassing model of evolution. After all, organizational ecology describes the processes of selection that provide the context for the processes of variation studied in evolutionary economics. An obvious next step, in line with organizational ecology, is to conduct exit or mortality analyses. Even though the true date of exit of patents cannot be known (after all, this would require information on the use of the patent by the organization that holds this patent), we do acknowledge that–as suggested by one of our reviewers–the exit of patents can be approximated by using data on patent maintenance fees. Patent maintenance fees have to be paid at the USPTO after 3.5, 7.5, and 11.5 years (depending on the size of the entity that holds the patent, these fees range from $400 to $7,400). Failure to pay these fees will result in the expiration of the patent. With the digitalization of the USPTO’s patent data, these data have recently become available, and can now be used to approximate the exit (or death) of patents. As this data was not available to us during our research, we do not include these in our analysis. However, future work can examine this data, which will lead to a better representation of the ecological process of patent entry and exit.

## Supporting Information

S1 Appendix Description of biotechnology’s component technologies. (DOCX)
