# Supplementary material for: The Population Ecology of Technology: An Empirical Study of US Biotechnology Patents from 1976 to 2003
Source: PLoS One. 2017 Jan 12;12(1):e0169961. doi: 10.1371/journal.pone.0169961 (PMC5233424; doi:10.1371/journal.pone.0169961)
Supplement: S1 Appendix — (DOCX) [file pone.0169961.s001.docx]

| **Component** | **Description** |
| --- | --- |
| 435001 | Differentiated tissue or organ other than blood, per se, or differentiated tissue or organ maintaining |
| 435002 | Maintaining blood or sperm in a physiologically active state or compositions thereof or therefor or methods of in vitro blood cell separation or treatment |
| 435003 | Condition responsive control process |
| 435004 | Measuring or testing process involving enzymes or micro-organisms |
| 435005 | Micro-organism, tissue cell culture or enzyme using process to synthesize a desired chemical compound or composition |
| 435006 | Process of mutation, cell fusion, or genetic modification |
| 435007 | Treatment of micro-organisms or enzymes with electrical or wave energy (e.g., magnetism, sonic waves, etc.) |
| 435008 | Carrier-bound or immobilized enzyme or microbial cell |
| 435009 | Enzyme (e.g., ligases (6. ), etc.), proenzyme |
| 435010 | Virus or bacteriophage, except for viral vector or bacteriophage vector |
| 435011 | Animal cell, per se (e.g., cell lines, etc.) |
| 435012 | Plant cell or cell line, per se (e.g., transgenic, mutant, etc.) |
| 435013 | Spore forming or isolating process |
| 435014 | Micro-organism, per se (e.g., protozoa, etc.) |
| 435015 | Vector, per se (e.g., plasmid, hybrid plasmid, cosmid, viral vector, bacteriophage vector, etc.) bacteriophage vector, etc.) |
| 435016 | Process of utilizing an enzyme or micro-organism to destroy hazardous or toxic waste, liberate, separate, or purify a preexisting compound or composition therefore |
| 435017 | Apparatus |
| 435108 | Miscellaneous (e.g., subcellular parts of micro-organisms, etc.) |
| 800001 | Method of using a transgenic nonhuman animal in an in vivo test method (e.g., drug efficacy tests, etc.) |
| 800002 | Method of using a transgenic nonhuman animal to manufacture a protein which is then to be isolated or extracted |
| 800003 | Nonhuman animal |
| 800004 | Method of making a transgenic nonhuman animal |
| 800005 | Method of using a plant or plant part in a breeding process which includes a step of sexual hybridization |
| 800006 | Method of chemically, radiologically, or spontaneously mutating a plant or plant part without inserting foreign genetic material therein |
| 800007 | Method of producing a plant or plant part using somatic cell fusion (e.g., protoplast fusion, etc.) |
| 800008 | Method of introducing a polynucleotide molecule into or rearrangement of genetic material within a plant or plant part |
| 800009 | Plant, seedling, plant seed, or plant part, per se |
